# Influence of Cognitive Neuroscience on Contemporary Philosophy of Science

**DOI:** 10.1515/tnsci-2019-0007

**Published:** 2019-04-23

**Authors:** Fang Ren

**Affiliations:** 1 Xi’an Jiaotong University, Xi’an 710049, China

**Keywords:** Cognitive neuroscience, Contemporary philosophy of science, Implications

## Abstract

The study of contemporary philosophy of science based on cognitive neuroscience has strongly promoted the philosophy study of brain cognitive problems. It has pointed out the research direction for human to explore the relationship between the traditional mind and brain while systematically reflecting and investigating the theoretical basis and research method of cognitive neuroscience. Therefore, this study explores the influence and the significance of cognitive neuroscience on contemporary philosophy of science.

## 1 Introduction

Cognitive science is an emerging interdisciplinary subject including cognitive psychology, artificial intelligence, philosophy, linguistics, anthropology and cognitive neuroscience. Its birth and development have had a profound impact on contemporary philosophy of science. Cognitive science is an emerging interdisciplinary subject involving cognitive psychology, artificial intelligence, philosophy, linguistics, anthropology, and cognitive neuroscience. Its birth and development have had a profound impact on contemporary philosophy of science. The study of the formation of scientific theory from the perspective of cognitive psychology has become a new trend in contemporary philosophy of science. Cognitive psychologists generally oppose the practice of logical positivism and behaviourism against psychology, emphasizing the study of human psychological processes, and treating cognitive processes as a series of information processing that can symbolize external environmental events and themselves. Knowledge representation, problem solving and reasoning, pattern recognition, memory learning, and language problems are not only important research topics in psychology, but also issues in the study of philosophy of science. The knowledge, concepts, and thinking of cognitive psychology research provide a new perspective for scientific philosophers to solve the problem of philosophy of science.

In the 1980s, cognitive scientists realized that neuroscience was the foundation of cognitive science. Because the brain’s thinking process was not clarified, artificial brain simulation of the brain was fundamentally impossible. Functionalism’s view of studying the mind without relying on the brain is increasingly in trouble, and cognitive scientists are paying more attention to the research of neuroscience. After the emergence of cognitive neuroscience in the 1990s, cognitive neuroscience is a new-born that combines the compelling cognitive science and neuroscience in scientific development. It is not only loved by both parents, but also by computers. The concern of the scientific community, because the thorny problems encountered in intelligent computer research are all related to cognitive neuroscience. The research task of cognitive neuroscience is to clarify the brain mechanism of cognitive activity. In other words, how does the human brain call it? The various levels of components, including molecules, cells, brain tissue regions, and brains to achieve their own cognitive activities are fundamental propositions that cognitive neuroscience needs to answer.”

The integration of various disciplines provides support for the study of contemporary philosophy of science which aims at “clarifying the brain mechanism of cognitive activities, namely, how the human brain mobilizes its components at all levels, including molecules, cells, brain regions and the whole brain, to achieve various cognitive activities” ^[1]^. The traditional ability of solving epistemological problems is not enough to reveal the riddle of human cognition, so it is necessary to discuss and construct the mechanism, model and reason of human cognition in an empirical way and a naturalistic way from the inside of cognitive science. Thus, the derived contemporary philosophy of science based on cognitive neuroscience has become the hottest research field of philosophy of science at present ^[2]^. Therefore, this study explores the influence and the significance of cognitive neuroscience on contemporary philosophy of science.

### 1.1 Introduction to cognitive neuroscience

The study of the formation of scientific theory from the perspective of cognitive psychology has become a new trend in contemporary philosophy of science. Cognitive psychologists generally oppose the practice of logical positivism and behaviourism against psychology, emphasizing the study of human psychological processes, and treating cognitive processes as a series of information processing that can symbolize external environmental events and themselves process. Knowledge representation, problem solving and reasoning, pattern recognition, memory learning, and language problems are not only important research topics in psychology, but also issues in the study of philosophy of science. The knowledge, concepts, and thinking of cognitive psychology research provide a new perspective for scientific philosophers to solve the problem of philosophy of science. Scientific philosophers began to draw on the research results of cognitive psychology. For example, Kuhn used psychology’s Gestalt to transform the paradigm shift of analogical science theory. Gonick applied the results of human individual cognitive development of cognitive psychology to scientific theory. The evolution of Nassian explains the development of physics theory from the perspective of cognitive psychology. Jill studies the cognitive structure of scientific theory from the perspective of psychology. As Sagard said that at present, the philosophy of science has undergone a cognitive shift, and it attempts to study the development of science from the perspective of cognitive psychology and artificial intelligence.

**Figure 1:**
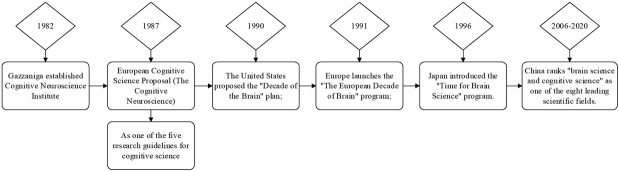
The development of cognitive neuroscience

Cognitive neuroscience is a discipline to study cognition from the level of cranial nerve, and is an important field of learning science. It mainly focuses on the neural mechanisms of perception, selective attention, memory, language, emotion and consciousness. Cognitive neuroscience has a history of nearly 20 years. It began to come into people’s view in the 1980s and became a new branch of science recognized by international academic circles in the 1990s. Its development history is shown in Figure 1.

Cognitive neuroscience belongs to a branch of the field of brain structure and function science, which is highly interdisciplinary and trans-disciplinary. Cognitive neuroscience attaches great importance to rigorous experimental research and the application of scientific research methods and technologies. The emergence of various brain function image technology has laid a solid methodological foundation for the rapid development of cognitive neuroscience ^[4]^. The main research methods and technologies are shown in Table 1.

**Table 1:** Main methods and techniques of cognitive neuroscience

Event-related brain potential (ERP)	A series of brain waves formed by a brain reaction that has a fixed time relationship with actual or expected stimuli (sound, light, and electricity).
Positron Emission Tomography (PET)	Using a large number of radioactive isotopes present in the natural elements of the human body, multi-level cross-sectional imaging is performed to reflect static or dynamic brain functional status.
fMRI	Similar to PET, changes in cerebral blood flow and deoxygenated haemoglobin concentrations can be recorded by fMRI to indirectly study neuronal function

### 1.2 Current status and development trend of contemporary philosophy of science

Cognitive science makes cognitive problems the focus of scientific philosophy research. Logical empiricism and falsifications focus on the logical structure of scientific knowledge. Historicism pays attention to the social and historical investigation of scientific knowledge, while ignoring the essence of science and the cognitive phenomenon of the core. The philosopher of science always pays attention to the objective content reflected by scientific theory and ignores the relationship between this content and the cognitive subject. Cognitive science requires studying the cognitive ability of the examiner to study the role of the individual’s internal cognitive factors in forming scientific theories. Scientific philosophers must not only analyse linguistic, logical, historical, and cultural sciences, but also conduct cognitive analysis. The combination of cognitive analysis and linguistic analysis, logical analysis, historical analysis and cultural analysis reinforces the cognitive function of scientific philosophy. For example, Sagad combines cognitive and social factors to study new models of science, and illustrates its effectiveness through case studies of the chemical revolution.

Nowadays, philosophy of science is in a “non-breakthrough” period as a whole, that is, there is no major breakthrough theory. However, no matter how the philosophy of science develops, the core of its scientific methodology can’t be changed for the reasons shown in Table 2.

**Table 2:** Reasons for not changing the scientific methodology of contemporary philosophy of science

No.	Reason
1	Scientific rationality cannot be dispelled, and philosophy of science should always hold high the banner of scientific rationality.
2	The philosophical problems of natural science cannot be dispelled. This is the foundation on which the philosophy of science depends.
3	The analysis method of language philosophy and its context theory are the basis of the various schools of unified philosophy of science and their traditional methodology.
4	The subject of science cannot replace scientific questioning methods with social, epistemological, and psychological things.

At present, pluralism is standing on the standpoint of cultural analysis to appeal to the cultural explanation of scientific development. Although this explanation expands the perspective and scope of contemporary philosophy of science on a certain level, it has the tendency of pan-culturalism and the tendency of dispelling scientific rationality. The philosophy of technology itself is inherently endowed with more cultural traits

that determine that it is not based on the requirements of pure scientific rationality ^[5]^. In the 20th century, the cognition, understanding and exploration of these problems are a process from nature to necessity. Through reflection on the course of the 20th century, the development of contemporary philosophy of science towards the 21st century has formed the main objectives and “leading difficulties”, as shown in Table 3.

**Table 3:** The main objectives of contemporary philosophy of science and its guiding difficulties”

Main target	Difficulty
Rebuilding the New Logical Starting Point for the Development of Philosophy of Science	How does the starting point surpass the paradigm of logical empiricism, historicism, and post-historicism
Build a new platform for mutual dialogue, exchange, penetration and integration between scientific realism and anti-realism among various schools.	How to do this on the platform, they can truly communicate with each other and promote together, making it a stage for the growth of scientific philosophy.
Exploring a new base for the mutual reference, mutual complementarity, and cross-intersection of various scientific methodologies	On this basis, how to obtain an effective unity of the philosophy of science and forge a viable innovation theory and development direction
Adhere to the essence of scientific rationality; we must continue to promote the spirit of scientific rationality.	Only on this basis can we talk about the unity of scientific rationality and irrationality, and talk about the connection between scientific philosophy and the schools or disciplines of scientific sociology, scientific knowledge theory, scientific history, and scientific culture and philosophy.

## 2 The influence of cognitive neuroscience on the philosophy of science

The development trend of cognitive neuroscience is from paying attention to the neurobiological activities of the brain to paying attention to the high-level cognitive function of the brain; from focusing on attention to partial level to focusing on the whole brain level; from paying attention to the relationship between brain activity and behaviour to paying attention to the development of brain structure and function; from attaching importance to brain and external behaviour to attaching importance to “gene and environment—brain—behaviour”. Based on the theoretical content of contemporary philosophy of science (as shown in Table 4) this part describes the influence of cognitive neuroscience on contemporary philosophy of science.

**Table 4:** Theoretical content of contemporary philosophy of science

Content	Manifestations
Science as a knowledge production activity	Scientific practice reflects nature, clearly shows the way in which science is presented, and highlights the ways and methods for discovering science under social and historical circumstances.
Science as a knowledge utilization activity	Using science as a paradigm to solve difficult problems using scientific knowledge
Science as a technology transformation activity	Reflecting the movement of the material world and the development of change in the form of truth, it is the main means and method for humans to understand truth and falsehood.

### 2.1 Cognitive neuroscience influences the cognitive turn of contemporary philosophy of science

Human culture influences the humanistic trend of philosophy of science. Human cultural scientists focus on the impact of environment and culture on cognitive ability, and regard cognition as a cultural phenomenon. According to Leslie A. White’s research, human intelligence has hardly developed in thousands of years, and cognitive development is the result of culture. All human culture, including science, relies on symbols, culture, not society. It is a distinctive characteristic of human beings. Culture has a more direct and more important role in science than science in society; a discovery and invention is a synthesis of cultural elements that have existed or a new element is absorbed into one in a cultural system. Maurice N. Richter (Jr) believes that science is a cultural counterpart as a cognitive development of an individual, a growth of traditional cultural knowledge, and a cognitive form of cultural development; The direction of development is similar to the direction of individual cognitive development. The starting point of scientific development is traditional cultural knowledge. The structure of scientific development is generally similar to the structure of evolutionary process, especially the structure similar to the process of cultural evolution. Science is an individual. The extension of the level to the cognitive development of the cultural level, it is not only a developmental growth above the traditional cultural knowledge, but also a special cognitive variant and extension of cultural evolution. Brune Latour and Karin D. Knorr - Cetina used anthropological methods to study the actual cognitive processes of scientists in the laboratory. Xie Tingna called this method a micro-prone method. Their research shows that the process of scientific cognition is the process by which experimental personnel create scientific facts. Scientific experiments are not to discover facts but to create facts. The experimental social network is not a scientific community, but a social network formed by super-recognizing resource relationships. Cultural studies of science have moved the philosophy of science from a philosophy of science to a philosophy of science and culture. Sixth, cognitive neuroscience affects the cognitive shift of philosophy of science. Qiu Qilande believes: “The scientific philosopher is a cognitive scientist. For a long time, philosophers are the only cognitive scientists.” As philosophers of cognitive scientists, they did not initially consider neuroscience as cognitive science. In part, and the whole philosophical foundation of artificial intelligence is considered to be anti-biological, it is advocated that the abstract program hierarchy with algorithms is independent of the neurobiology of the brain or the hardware level of the computer silicon chip. In other words, neurobiological details do not help people understand the level of cognition.

Philosophers, as cognitive scientists, did not regard neuroscience as part of cognitive science at first, and believed that the entire philosophical basis of artificial intelligence is anti-biological; advocating that the abstract program level with algorithms was independent of the neurobiology of the brain or the hardware level of a computer silicon chip. All the difficult problems encountered in intelligent computer research are related to cognitive neuroscience. The task of cognitive neuroscience is to clarify the brain mechanism of cognitive activities. The discussion of the status quo and future of artificial intelligence has already become the focus of popular culture, which will have far-reaching influence on the development of the organization form and structure of the whole human society ^[7]^. According to the structure of cognitive neuroscience and contemporary philosophy of science, the artificial intelligence can be divided into three levels: artificial intelligence, artificial life and artificial wisdom based on cognitive dimension and philosophy of science (as shown in Table 5).

**Table 5:** Artificial intelligence hierarchy

Artificial intelligence level	Division basis	Thinking qualities	Functional positioning
Artificial intelligence	Have smart behaviour or performance	Mainly based on memory level	Extension of human thinking and functional assistant
Artificial life	Self-reproducing ability	Have symbol communication skills	Different from human primary life
Artificial intelligence	Self-awareness and emotion	Emotional thinking ability	May be dangerous to humans and need special treatment

### 2.2 Human culture influences the humanistic trend of contemporary philosophy of science

All human cultures, including science, depend on symbols. It is culture rather than society that is the distinctive feature of human beings. Culture plays a more direct and important role in science than society. A kind of discovery and invention is the synthesis of existing cultural elements or the absorption of a new element into a cultural system ^[8]^. The direction of scientific development is similar to that of individual cognitive development. The starting point of scientific development is traditional cultural knowledge. The structure of scientific development is generally similar to that of evolutionary process. It is not only the growth of traditional cultural knowledge, but also a special cognitive variant and extension of cultural evolution. Through the accumulation of traditional cultural knowledge, contemporary philosophers of science can provide basic concepts and ideological basis for cognitive neuroscience, as well as examine and reflect on the basic assumptions by using the methods of argument, conceptual analysis and historical reflection (as shown in Table 6).

**Table 6:** The philosophical method of the use of cognitive neuroscience by contemporary philosophers of science

Methods	Manifestations
Dispute method	Contemporary scientific philosophy penetrates into the core of various disciplines with its dialectical role, and dialectically analyses the contradictions between scientists’ empirical findings and theories.
Concept description method	The concept of cognition plays an important role in cognitive science. The concept of cognition is the premise and foundation of cognition and reasoning. In cognitive science, cognitive science tends to focus on the specific process of acquiring new concepts from experience or other concepts.
Historical reflection and critical method	Philosophical reflection can transcend experience, exceed existing, present and present, and overcome common sense and prejudice. The exploratory and creative nature of philosophical activities and the charm of philosophy are reflected in this critical reaction of “making problems a problem”.

### 2.3 Modern philosophy of mind provides cognitive theoretical basis for contemporary scientific philosophers to explore neurocognitive process

Cognitive science deepens the understanding of the nature of scientific knowledge. Logical empiricism regards scientific knowledge as a static propositional language system, falsifications emphasizes the process of conjecture of scientific knowledge acquisition, and historicism exaggerates the social agreement factors formed by scientific knowledge. They submerged cognitive problems in language analysis, historical analysis and psychosocial analysis, replacing cognition with the linguistic, historical and social psychology of scientific knowledge, ignoring the close correlation between scientific knowledge and psychological representation. Although the early thinkers Plato, Locke, and Kant discussed the psychological representation, they were rejected by positivism and behaviourism as a metaphysical construction. The development of cognitive science has made people realize that scientific knowledge is the representation and symbolization of the real world in psychology, and psychological representation provides a partial picture of the real world. Scientists form a psychological representation of all aspects of their activities, so that scientific knowledge is reflected in the psychological representation of scientists. The current central hypothesis of cognitive science holds that the most appropriate understanding of thinking is to regard it as a representational structure in the mind and a computational program that operates on these structures. People generate thoughts and behaviours by running psychological programs on top of psychological representations, while different types of psychological representations of logic, rules, concepts, analogies, representations, and connections support different types of psychological procedures. These characterizations together constitute the scientist’s knowledge system, and through the communication and dialogue between scientists, form a common representation of the scientific community. Therefore, psychological

representations represent the essence of scientific theory, and the theory is hidden in the psychological representation of scientists.

**Figure 2:**
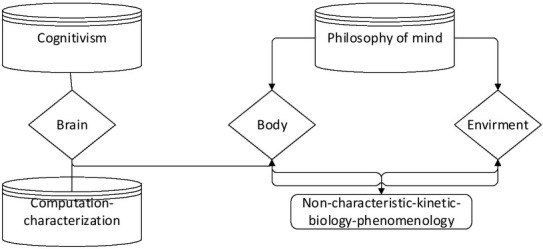
The basic style of cognitivism and philosophy of mind

The philosophy of mind with information processing as its core has exerted an important influence on scientific realism. To some extent, both enactive cognition and situational cognition can be integrated by embodied cognition ^[9]^. Therefore, from the discussion of these two points, we can basically distinguish between cognitivism and embodied cognition approach (as shown in Figure 2).

## 3 The significance of cognitive neuroscience to the philosophy of science

The emergence of cognitive scientists with philosophical minds and philosophers with cognitive science knowledge is very useful to overcome this gap. As mentioned above, the profound influences of cognitive neuroscience on contemporary philosophy of science will cause fundamental changes in the research strategy of philosophy of science and the basis of science of interpretation, as shown in Table 7. Based on this, the significance of cognitive neuroscience to contemporary philosophy of science is expressed in the following four aspects:

**Table 7:** The Change of Cognitive Neuroscience to Contemporary Philosophy of Science

Cognitive neuroscience makes cognitive issues the focus of contemporary scientific philosophy.	The combination of cognitive analysis and language analysis, logical analysis, historical analysis and cultural analysis reinforces the cognitive function of philosophy of science.
Cognitive neuroscience deepens the understanding of the nature of scientific knowledge.	Think of it as the characterization structure in the mind and the computational procedures that operate on these structures
Cognitive neuroscience contributes to the solution of problems found in contemporary philosophy of science.	The discovery of contemporary philosophy of science is a solution to creative problems and is a sequence of cognitive operations guided by goals.
Cognitive Neuroscience Deepens the Evaluation of Contemporary Philosophy of Science	Cognitive neuroscience puts the evaluation of the philosophy of science between the individual’s selection criteria and the objective criteria commonly recognized by the scientific community.

### 3.1 Providing basic concepts, ideological foundations and reflections for contemporary philosophy of science

The most basic thought of cognitive science comes from the construction of philosophy. The task of cognitive neuroscience is to clarify the brain mechanism of cognitive activities ^[10]^. People produce thinking and behaviour by running psychological procedure on mental representation while logic, rule, concept, analogy, representation and connection of mental representation support different types of philosophical forms. These representations constitute the knowledge system of the contemporary philosophers of science, and form the common representation of the contemporary scientific philosophy community through the communication and dialogue between the scientific philosophers. To this end, we must reflect on the problems as shown in Table 8.

**Table 8:** Reflection on contemporary philosophy of science under cognitive neuroscience

The Origin Reflection of Contemporary Philosophy of Science Based on Cognitive Neuroscience.	Thinking about the general research ideas and assumptions followed by cognitive neuroscience is a set of rigorous and orderly natural science research ideas and research hypotheses, and it is also a science-based armed force that psychology pursues science.
Methodological Reflection on Contemporary Philosophy of Science in Cognitive Neuroscience.	Scientific methods determine the choice of scientific research issues and determine the appropriateness of scientific knowledge. The progress of science depends on the progress and improvement of scientific methods.

### 3.2 Examining and reflecting the basic assumption of contemporary philosophy of science

For cognitive neuroscience, each specialized theory that explores the mind is assumed based on basic theory and methodology, and forms the basis of the entire cognitive neuroscience despite further testing is needed. The basic assumption of dualism is that the mind exists independently of the body while the basic assumption of materialism is that the mind depends on living body. The basic assumption of behaviourism is that the mind doesn’t exist and the behaviour determines the function while the basic assumption of functionalism is that the psychological state and the physical state are causal relations. The assumption of representational computation is that the mind is a process of representational computation.

### 3.3 Clarify and test the basic issues and propositions of contemporary philosophy of science

In cognitive neuroscience, contemporary philosophers of science usually don’t conduct systematic empirical observations or build computational models. They deal with some basic problems of cognitive neuroscience, such as the rationality of computer metaphor, the nature of representation and computation, general knowledge, the nature of intelligence, and the essence of explanation in cognitive science, the function of consciousness in mental research, how people carry out normative thinking, so as to clarify these questions and test the certainty from the logic and significance. Therefore, we can say that contemporary scientific philosophy without cognitive neuroscience is empty, and cognitive neuroscience without contemporary scientific philosophy is blind.

## 4 Conclusions

These profound influences of cognitive science on philosophy of science will fundamentally change the research strategy of science philosophy and the foundation of science. Cognitive science makes cognitive problems the focus of scientific philosophy research. Cognitive science requires studying the cognitive ability of the examiner to study the role of the individual’s internal cognitive factors in forming scientific theories. The combination of cognitive analysis and linguistic analysis, logical analysis, historical analysis and cultural analysis reinforces the cognitive function of scientific philosophy.

The flourishing research of contemporary neuroscience and cognitive neuroscience has not only answered and solved the mechanism of human cognitive function, but also enriched and supported the research content of philosophy of cognitive neuroscience. Neuroscience with neuron and brain structure as its research object is connected with philosophy, especially philosophy of science, which has become a frontier field in the development of contemporary philosophy of science. From the perspective of philosophy, especially epistemology, the emergence of the philosophy of cognitive neuroscience is an inevitable process of exploring the mechanism of human cognition to a great extent, which obviously becomes the direct impetus of the emergence of cognitive neuroscience in the field of contemporary philosophy of science.

## References

[1] Schaal D. W. (2013). Naming our concerns about neuroscience: a review of bennett and hacker’s philosophical foundations of neuroscience. Journal of the Experimental Analysis of Behavior.

[2] Georgieff N. (2011). Psychoanalysis and social cognitive neuroscience: a new framework for a dialogue. J Physiol Paris.

[3] Dwight D. (2015). The measure of madness: philosophy of mind, cognitive neuroscience, and delusional thoughtby philip gerrans.

[4] Frisch S. (2014). How cognitive neuroscience could be more biological—and what it might learn from clinical neuropsychology. Frontiers in Human Neuroscience.

[5] Hyett M. P. (2010). Contemporary debates in cognitive science. Acta Neuropsychiatrica.

[6] Mccall B. (2011). Contemporary debates in cognitive science (contemporary debates in philosophy). edited by robert j. stainton and cognitive integration: mind and cognition unbounded (new directions in philosophy and cognitive science). by richard menary. Heythrop Journal.

[7] Jin-Mei L. I., Zhi-Yong Z. (2013). Interpretation of construal of ci text from the perspective of embodied conceptualization of cognitive linguistics. Journal of Qiqihar University.

[8] Gelder V Tim (1998). The roles of philosophy in cognitive science. Philosophical Psychology.

[9] Nicolás Venturelli A. (2016). A cautionary contribution to the philosophy of explanation in the cognitive neurosciences. Minds & Machines.

[10] Fox N., Marshall G. P., Ghera M. M., Henderson J., Nichols T. E. (2005). Behavioral inhibition: linking biology and behavior within a developmental framework. Annual Review of Psychology.

[11] Forstmann B. U., Ratcliff R., Wagenmakers E. (2015). Sequential sampling models in cognitive neuroscience: advantages, applications, and extensions. Annual Review of Psychology.

[12] Meyering T., C. (1996). Philosophical psychology in historical perspective: review essay of j.‐c. smith (ed.), historical foundations of cognitive science. Philosophical Psychology.

